# Supplementary material for: Content-rich biological network constructed by mining PubMed abstracts
Source: BMC Bioinformatics. 2004 Oct 8;5:147. doi: 10.1186/1471-2105-5-147 (PMC528731; doi:10.1186/1471-2105-5-147)
Supplement: Additional File 2 — The original results of the above study (non-essential files are deleted to keep the file size under the limit set by BMC bioinformatics). [file 1471-2105-5-147-S2.bz2 › chilibotAdditionalFile2/dip50/497ID10187774E10660/html/TGFB1_ACVRL1.html]

 


 **TGFB1** and **ACVRL1** 
  
Found 99 abstracts in PubMed, retrieved 50.  
 

 What does Google say? 
 PDF only 
| .edu only 

---

**Interactive relationship** (e.g. stimulation, inhibition, etc)

**Inhibitory relationship**- The blood vessel defects in ALK1  [ **ACVRL1** ]  deficient mice are reminiscent of mice lacking TGF beta1  [ **TGFB1** ] , TGF beta  [ **TGFB1** ]  type II receptor TbetaR II, or endoglin, suggesting that ALK1  [ **ACVRL1** ]  may mediate TGF beta1  [ **TGFB1** ]  signal in endothelial cells.  Ref: 10716993 Proc Natl Acad Sci U S A, 2000
- Furthermore, the ALK1  [ **ACVRL1** ]  signaling pathway can inhibit TGF beta1  [ **TGFB1** ]  dependent transcriptional activation mediated by the known TGF beta1  [ **TGFB1** ]  type I receptor, ALK5.  Ref: 10716993 Proc Natl Acad Sci U S A, 2000
- Stable expression of these alternate ALK  [ **ACVRL1** ]  5 isoforms in ALK  [ **ACVRL1** ]  5 deficient cells demonstrated that both were competent in signaling TGF beta  [ **TGFB1** ]  induced growth inhibition and gene transcription, but with an apparently distinct potency.  Ref: 10664470 FEBS Lett, 2000
- LXRbeta subsequently modulates signaling by ALK 1  [ **ACVRL1** ]  and the closely related TGF beta  [ **TGFB1** ]  receptor, ALK  [ **ACVRL1** ]  2, as demonstrated by specific and potent inhibition of ALK 1  [ **ACVRL1** ]  and ALK  [ **ACVRL1** ]  2 mediated transcriptional responses, establishing LXRbeta as a potential modulator mediator of ALK 1  [ **ACVRL1** ]  ALK  [ **ACVRL1** ]  2 signaling.  Ref: 12393874 J Biol Chem, 2002
**Neutral relationship**- Endoglin is a bifunctional receptor partner since it can bind to ALK 1  [ **ACVRL1** ]  as well as to type I TGF beta  [ **TGFB1** ]  receptor.  Ref: 10187774 J Biol Chem, 1999
- Transforming growth factor beta TGF beta  [ **TGFB1** ]  complex has been implicated in HHT  [ **ACVRL1** ] .  Ref: 10235000 Neurol Med Chir Tokyo , 1998
- Activin receptor like kinase 1  [ **ACVRL1** ]  modulates transforming growth factor beta 1  [ **TGFB1** ]  signaling in the regulation of angiogenesis.  Ref: 10716993 Proc Natl Acad Sci U S A, 2000
- ALK  [ **ACVRL1** ]  5 and ALK 1  [ **ACVRL1** ]  mediate TGF beta  [ **TGFB1** ]  signals through distinct Smad proteins, i.e., Smad2 Smad3 and Smad1 Smad5, respectively.  Ref: 12384983 J Cell Physiol, 2002
- **ACVRL1**  encodes a type I receptor for the TGF beta  [ **TGFB1** ]  superfamily of growth factors.  Ref: 11062473 Nat Genet, 2000
- Activin receptor like kinase 5 ALK  [ **ACVRL1** ]  5 is a TGF beta  [ **TGFB1** ]  type I receptor expressed in various TGF beta  [ **TGFB1** ]  responsive cells.  Ref: 12384983 J Cell Physiol, 2002
- TGF beta 1  [ **TGFB1** ]  bound to TGF beta  [ **TGFB1** ]  type I receptor T beta R I ALK  [ **ACVRL1** ]  5 and T beta R II, but not to activin type I receptor ActR I ALK  [ **ACVRL1** ]  2.  Ref: 10574705 J Cell Sci, 1999
- In COS 1 transfected cells, ALK 1  [ **ACVRL1** ]  was found in the TGF beta1  [ **TGFB1** ]  and beta3 receptor complexes in association with endoglin and TbetaRII, but not in activin receptor complexes containing endoglin.  Ref: 10767348 Hum Mol Genet, 2000
- ALK 1  [ **ACVRL1** ]  is an endothelial cell type I receptor for the TGF beta  [ **TGFB1** ]  superfamily of ligands.  Ref: 10694922 Hum Mutat, 1998
- The ALK 1  [ **ACVRL1** ]  ligand interaction is mediated by the type II TGF beta  [ **TGFB1** ]  receptor for TGF beta  [ **TGFB1** ]  and most likely through the activin type II or type IIB receptors for the serum ligand.  Ref: 10187774 J Biol Chem, 1999
- Endoglin and ALK 1  [ **ACVRL1** ]  are type III and type I TGF beta  [ **TGFB1** ]  receptors, respectively, and are exclusively expressed on vascular endothelial cells.  Ref: 11019486 J Med Invest, 2000
- The activin receptor like kinase 1  [ **ACVRL1** ]  ALK1  [ **ACVRL1** ]  is a type I receptor for transforming growth factor beta TGF beta  [ **TGFB1** ]  family proteins.  Ref: 10716993 Proc Natl Acad Sci U S A, 2000
- Mutations in ALK1  [ **ACVRL1** ] , a TGF beta  [ **TGFB1** ]  type 1 receptor, previously known to cause type 2 hereditary haemorrhagic telangiectasia HHT  [ **ACVRL1** ] , have also been reported in a few HHT  [ **ACVRL1** ]  families with clinical and histological features of PPH.  Ref: 11817654 Ann Med, 2001
- Transfection of constitutively active CA ALK  [ **ACVRL1** ]  4 activated the 3TP promoter to the same extent as TGF beta 1  [ **TGFB1** ]  and CA ALK  [ **ACVRL1** ]  5 indicating that activin signalling downstream of type I receptors was functional in NMuMG cells.  Ref: 10574705 J Cell Sci, 1999
- Our results suggest that TGF beta  [ **TGFB1** ]  regulates the activation state of the endothelium via a fine balance between ALK5 and ALK1  [ **ACVRL1** ]  signalling.  Ref: 11927558 EMBO J, 2002
- In endothelial cells, TGF beta  [ **TGFB1** ]  binds to two distinct type I receptor serine threonine kinases, ALK  [ **ACVRL1** ]  5 and ALK 1  [ **ACVRL1** ] .  Ref: 12297674 Sci STKE, 2002
- These data suggest that modifier genes involved in the regulation of TGF beta1  [ **TGFB1** ]  expression act in combination with a single functional copy of endoglin in the development of HHT  [ **ACVRL1** ] .  Ref: 11395379 Am J Pathol, 2001
- Recent studies have identified mutations of the bone morphogenetic protein receptor 2 BMPR2 gene and the activin receptor like kinase 1  [ **ACVRL1** ]  ALK1  [ **ACVRL1** ]  gene, that affect the transforming growth factor beta TGF beta  [ **TGFB1** ]  receptor superfamily, a group of transmembrane signaling molecules with serine threonine kinase activity that are involved in the regulation of cell growth.  Ref: 12624796 Z Rheumatol, 2003
- Recently, mutations in BMPR2 and ALK 1  [ **ACVRL1** ] , genes that encode members of the transforming growth factor beta TGF beta  [ **TGFB1** ]  receptor superfamily, have been found in patients with primary pulmonary hypertension.  Ref: 11869166 Clin Sci Lond , 2002
- Bone morphogenetic protein IB BMP IB receptor also known as ALK  [ **ACVRL1** ]  6, which binds members of the transforming growth factor beta TGF beta  [ **TGFB1** ]  superfamily, is located in the region containing the FecB locus.  Ref: 11259271 Biol Reprod, 2001
- ALK 1  [ **ACVRL1** ]  activin receptor like kinase 1  [ **ACVRL1** ] , a type I receptor of the transforming growth factor TGF beta  [ **TGFB1** ]  superfamily, is the gene mutated in hereditary hemorrhagic telangiectasia type 2 HHT2  [ **ACVRL1** ]  while endoglin is mutated in HHT1.  Ref: 10767348 Hum Mol Genet, 2000
- Activin receptor like kinase 1  [ **ACVRL1** ]  ALK 1  [ **ACVRL1** ] , the gene mutated in HHT  [ **ACVRL1** ]  type 2 HHT2  [ **ACVRL1** ] , is a serine threonine kinase receptor type I of the TGF beta  [ **TGFB1** ]  superfamily, specifically expressed on endothelial cells.  Ref: 12700602 Eur J Hum Genet, 2003
- In contrast, ALK 1  [ **ACVRL1** ]  functions as a TGF beta  [ **TGFB1** ]  type I receptor in endothelial cells, and is responsible for human hereditary hemorrhagic telangiectasia HHT  [ **ACVRL1** ]  type II.  Ref: 12384983 J Cell Physiol, 2002
- The genetic cause of this disease lies in different defects of transmembrane proteins endoglin, activin receptor like kinase 1  [ **ACVRL1** ]  that can be defined as components of the receptor complex for transforming growth factor beta TGF beta  [ **TGFB1** ] .  Ref: 11505335 Z Gastroenterol, 2001
- Immunohistochemistry demonstrated that, compared to controls, shunt lambs have increased TGF beta1  [ **TGFB1** ]  and TbetaRI expression in the smooth muscle layer, and increased TGF beta1  [ **TGFB1** ]  and ALK 1  [ **ACVRL1** ]  in the endothelium of small pulmonary arteries < ; 200 um at 1 and 4 weeks of age.  Ref: 12665463 Am J Physiol Lung Cell Mol Physiol< MedlineTA>Am J Physiol Lung Cell Mol Physiol, 2001

**Non-interactive relationship** (e.g. studied together, co-existance, homology, etc.)

- The TGF beta  [ **TGFB1** ]  type I receptor ALK  [ **ACVRL1** ]  5 and its effector Smad proteins mediate the epithelial to mesenchymal transition.  Ref: 10574705 J Cell Sci, 1999
- TGF beta  [ **TGFB1** ]  type I receptor ALK  [ **ACVRL1** ]  5 and Smad proteins mediate epithelial to mesenchymal transdifferentiation in NMuMG breast epithelial cells.  Ref: 10574705 J Cell Sci, 1999
- Binding of TGF beta  [ **TGFB1** ]  to the type II TGF beta  [ **TGFB1** ]  receptor on endothelial cells, which is accelerated in the presence of endoglin, phosphorylates type I TGF beta  [ **TGFB1** ]  receptors, ALK  [ **ACVRL1** ]  5 and ALK 1  [ **ACVRL1** ] , and phosphorylated ALK  [ **ACVRL1** ]  5 and ALK 1  [ **ACVRL1** ]  activate the downstream proteins Smad2 3 and Smad1 5, respectively.  Ref: 11019486 J Med Invest, 2000
- TGF beta  [ **TGFB1** ]  induces phosphorylation of Smad1 5 and Smad2 in endothelial cells and these effects can be blocked upon selective inhibition of ALK1  [ **ACVRL1** ]  or ALK5 expression, respectively.  Ref: 11927558 EMBO J, 2002
- In the two largest families, we used microsatellite markers to test for linkage to genes encoding TGF beta  [ **TGFB1** ]  receptor proteins, including endoglin and activin receptor like kinase 1  [ **ACVRL1** ]  ALK1  [ **ACVRL1** ] , and BMPR2.  Ref: 11484689 N Engl J Med, 2001
- Consistent with this hypothesis, we demonstrate that ALK1  [ **ACVRL1** ]  in endothelial cells binds to TGF beta1  [ **TGFB1** ]  and TbetaR II.  Ref: 10716993 Proc Natl Acad Sci U S A, 2000
- VEGF and its receptors flk 1 and flt 1 are the main actors of the morphogenic phase whereas PDGF BB and its receptor PDGF R beta, TGF beta 1  [ **TGFB1** ]  and its receptors ALK  [ **ACVRL1** ]  5 and ALK 1  [ **ACVRL1** ] , angiopoietin 1 and its receptor tie 2 are implied in the vascular maturation phase.  Ref: 10989550 Bull Acad Natl Med, 2000
- We show that TGF beta1  [ **TGFB1** ]  and TGF beta3, as well as a third unknown ligand present in serum, can activate chimeric ALK 1  [ **ACVRL1** ] .  Ref: 10187774 J Biol Chem, 1999
- The type I receptors examined were the TGF beta  [ **TGFB1** ]  type I receptor TbetaR I, activin type I receptor ActR I, activin type IB receptor ActR IB, bone BMP type IA receptor BMPR IA, BMP type IB receptor BMPR IB, and activin receptor like kinase 1  [ **ACVRL1** ]  ALK 1  [ **ACVRL1** ] .  Ref: 10321529 Acta Ophthalmol Scand, 1999
- Enalapril decreased renal TGF beta1  [ **TGFB1** ] , ALK 1  [ **ACVRL1** ] , and ALK  [ **ACVRL1** ]  5 protein expression P< ; 0.05.  Ref: 12883979 Pediatr NephrolPediatr Nephrol, 1999
- The recent discovery of germline mutations of genes coding for receptor members of TGF beta  [ **TGFB1** ]  BMPR 2 et ALK 1  [ **ACVRL1** ]  in PPH represents a considerable progress in the understanding of this pulmonary orphan disease.  Ref: 12134634 J Soc Biol, 2002
- Although ALK 1  [ **ACVRL1** ]  and another endothelial specific TGF beta  [ **TGFB1** ]  receptor, endoglin, both bind TGF beta  [ **TGFB1** ]  with identical isoform specificity and form a complex together, neither has been shown to signal in response to TGF beta  [ **TGFB1** ] , and the mechanism by which these receptors signal in endothelial cells remains unknown.  Ref: 12393874 J Biol Chem, 2002
- To understand the roles of TGF beta  [ **TGFB1** ]  isoforms TGF beta  [ **TGFB1** ]  and 3 and TGF beta  [ **TGFB1** ]  type I receptors activin receptor like kinase ALK  [ **ACVRL1** ] , 5 and 6 in PDL cell differentiation, their expression was investigated using Northern blot analysis.  Ref: 10633885 J Periodontal Res, 1999
- Our data suggest that alternate splicing within the ALK  [ **ACVRL1** ]  5 gene is an important mechanism whereby SMCs may regulate their response to TGF beta  [ **TGFB1** ] .  Ref: 10664470 FEBS Lett, 2000
- When the constitutively active form of the TGF beta  [ **TGFB1** ]  type I receptor ALK  [ **ACVRL1** ]  5 TD was ectopically expressed in osteoblastic cells, p57 Kip2 that had been accumulated by serum starvation causing the cell cycle arrest was rapidly degraded in a manner analogous to TGF beta1  [ **TGFB1** ]  stimulation.  Ref: 11152674 J Biol Chem, 2001
- We identified genes that are induced specifically by TGF beta  [ **TGFB1** ]  mediated ALK1  [ **ACVRL1** ]  or ALK5 activation.  Ref: 11927558 EMBO J, 2002
- We therefore studied the expression of UPA, UPAR, IGF2R, ALK5, TGFBR2, TGFBR3, ENG, ALK1  [ **ACVRL1** ] ,  **TGFB1** , TGFB2, and TGFB3 in a series of 14 pancreatic carcinoma cell lines.  Ref: 11408925 Int J Oncol, 2001
- TGF beta  [ **TGFB1** ]  type 1 receptor ALK  [ **ACVRL1** ]  5, activin type IB receptor ALK  [ **ACVRL1** ]  4, and the orphan ALK  [ **ACVRL1** ]  7 trans activate the Ialpha1 promoter, thus raising the possibility that other members of the TGF beta  [ **TGFB1** ]  superfamily can also modulate IgA synthesis.  Ref: 10652350 J Biol Chem, 2000
- In contrast, while the expression of type I receptor for TGF beta  [ **TGFB1** ]  ALK  [ **ACVRL1** ]  5 TbetaRI was detected, that of type II receptor TbetaRII was not.  Ref: 11078996 Eur J Endocrinol, 2000
- Id1 was found to mediate the TGF beta  [ **TGFB1** ]  ALK1  [ **ACVRL1** ]  induced and Smad dependent migration, while induction of plasminogen activator inhibitor 1 by activated ALK5 may contribute to the TGF beta  [ **TGFB1** ]  induced maturation of blood vessels.  Ref: 11927558 EMBO J, 2002
- Whereas the TGF beta  [ **TGFB1** ]  ALK5 pathway leads to inhibition of cell migration and proliferation, the TGF beta  [ **TGFB1** ]  ALK1  [ **ACVRL1** ]  pathway induces endothelial cell migration and proliferation.  Ref: 11927558 EMBO J, 2002
- To determine the potential role of TGF beta1  [ **TGFB1** ]  in this vascular remodeling, we then investigated vascular changes in expression and localization of TGF beta1  [ **TGFB1** ]  and its receptors TbetaRI, ALK 1  [ **ACVRL1** ] , and TbetaRII in the lungs of shunted and age matched control lambs at 1 day,, and 8 weeks of life.  Ref: 12665463 Am J Physiol Lung Cell Mol Physiol< MedlineTA>Am J Physiol Lung Cell Mol Physiol, 2002
- METHODS AND RESULTS The spatial relationships between TGF beta1  [ **TGFB1** ] , TGF beta3, ALK  [ **ACVRL1** ]  5, and TbetaR II expression were compared in aortic segments from 21 subjects.  Ref: 10359732 Circulation, 1999
- Alternative splicing within the TGF beta  [ **TGFB1** ]  type I receptor gene ALK  [ **ACVRL1** ]  5 generates two major functional isoforms in vascular smooth muscle cells.  Ref: 10664470 FEBS Lett, 2000
- No expression of TGF beta  [ **TGFB1** ]  type III receptor, endoglin, or the endothelial TGF beta  [ **TGFB1** ]  type I receptor ALK 1  [ **ACVRL1** ]  could be detected.  Ref: 10733095 J Leukoc Biol, 2000
- RESULTS TGF beta 1  [ **TGFB1** ]  mRNA did not colocalize with ALK  [ **ACVRL1** ]  5 mRNA.  Ref: 11318934 Kidney Int, 2001
- The TGFB  [ **TGFB1** ]  signalling associated receptors ENG and ALK1  [ **ACVRL1** ]  were co expressed in 4 of the cell lines.  Ref: 11408925 Int J Oncol, 2001
- Western blots demonstrated that TGF beta1  [ **TGFB1** ]  and ALK 1  [ **ACVRL1** ]  expression were elevated in the shunt as compared to control lambs at 1 and 4 weeks of age P< ; 0.05.  Ref: 12665463 Am J Physiol Lung Cell Mol Physiol< MedlineTA>Am J Physiol Lung Cell Mol Physiol, 2001
- These results indicate that ACE inhibition in the developing kidney decreases TGF beta1  [ **TGFB1** ] , ALK 1  [ **ACVRL1** ] , and ALK  [ **ACVRL1** ]  5 expression, which may account for renal growth impairment.  Ref: 12883979 Pediatr NephrolPediatr Nephrol, 2001
- Assignment of transforming growth factor beta1  [ **TGFB1** ]  and beta3 and a third new ligand to the type I receptor ALK 1  [ **ACVRL1** ] .  Ref: 10187774 J Biol Chem, 1999
- TGF beta1  [ **TGFB1** ] , ALK 1  [ **ACVRL1** ] , ALK  [ **ACVRL1** ]  5, and TbetaRII were also detected by immunohistochemistry.  Ref: 12883979 Pediatr NephrolPediatr Nephrol, 1999
- We thus demonstrate here that, while TbetaRI is crucial for the function of TGF beta  [ **TGFB1** ]  during vascular development and can not be compensated for by the activin receptor like kinase 1  [ **ACVRL1** ]  ALK 1  [ **ACVRL1** ] , functional hematopoiesis and development of hematopoietic progenitors is not dependent on TGF beta  [ **TGFB1** ]  signaling via TbetaRI.  Ref: 11285230 EMBO J, 2001
- This was accompanied by strong upregulation of the activin receptor ALK  [ **ACVRL1** ]  4 ActR IB and redistribution of the TGF beta  [ **TGFB1** ]  responsive ALK  [ **ACVRL1** ]  5 TbetaR I.  Ref: 11472976 Am J Respir Cell Mol Biol, 2001
- These data suggest that HHT  [ **ACVRL1** ]  pathogenesis involves disruption of a complex network of positive and negative angiogenic factors, involving TGF beta  [ **TGFB1** ] , a new unknown ligand, and their corresponding receptors.  Ref: 10187774 J Biol Chem, 1999
- In order to identify the ligand and type II receptor for ALK 1  [ **ACVRL1** ]  and to investigate the role of endoglin in ALK 1  [ **ACVRL1** ]  signaling, we devised a chimeric receptor signaling assay by exchanging the kinase domain of ALK 1  [ **ACVRL1** ]  with either the TGF beta  [ **TGFB1** ]  type I receptor or the activin type IB receptor, both of which can activate an inducible PAI 1 promoter.  Ref: 10187774 J Biol Chem, 1999
- Boston minipigs, treated with tranilast or vehicle, were subjected to endoluminal stenting, and the expression of TGF beta1  [ **TGFB1** ]  and TGF beta3, the expression of their signaling receptors ALK  [ **ACVRL1** ]  5 and TbetaR II, leukocyte numbers around the stent struts, and neointima development were assessed over 28 days.  Ref: 12067902 Arterioscler Thromb Vasc Biol, 2002
- This study was designed to investigate the relationship between renal growth impairment and the expression of transforming growth factor beta1  [ **TGFB1** ]  TGF beta1  [ **TGFB1** ] , TGF beta  [ **TGFB1** ]  receptor I TbetaRI, activin like kinase ALK 1  [ **ACVRL1** ]  and ALK  [ **ACVRL1** ]  5, and TGF beta  [ **TGFB1** ]  receptor II TbetaRII.  Ref: 12883979 Pediatr NephrolPediatr Nephrol, 2002
- Mutations of genes coding for TGF beta  [ **TGFB1** ]  receptors BMPR2 and ALK 1  [ **ACVRL1** ]  in primary pulmonary arterial hypertension < ArticleTitle>.  Ref: 12134634 J Soc Biol, 2002
- In contrast, constitutively active TGF beta  [ **TGFB1** ]  type I receptor, ALK  [ **ACVRL1** ]  5, did not exhibit any action on Smad8.  Ref: 10814522 Biochem Biophys Res Commun, 2000
- The transforming growth factor beta TGF beta  [ **TGFB1** ]  receptor, ALK 1  [ **ACVRL1** ] , is expressed specifically on endothelial cells and is essential for angiogenesis, as demonstrated by its targeted deletion in mice and its mutation in the human disease hereditary hemorrhagic telangiectasia.  Ref: 12393874 J Biol Chem, 2002
- Nonatherosclerotic intima contained predominantly TGF beta1  [ **TGFB1** ] , low concentrations of TbetaR II, and barely detectable amounts of ALK  [ **ACVRL1** ]  5.  Ref: 10359732 Circulation, 1999
- To distinguish between these possibilities in humans, immunohistochemical studies were performed examining the coexpression of TGF beta  [ **TGFB1** ]  isoforms and the TGF beta  [ **TGFB1** ]  receptors ALK  [ **ACVRL1** ]  5 and TbetaR II in aorta during the various stages of atherosclerotic lesion development.  Ref: 10359732 Circulation, 1999
- We have identified in rat vascular smooth muscle cells SMCs the simultaneous expression of two TGF beta  [ **TGFB1** ]  type I receptor ALK  [ **ACVRL1** ]  5 cDNAs, occurring as a consequence of alternate usage of AG splice acceptor motifs separated by 12 nucleotides located at an intron exon junction.  Ref: 10664470 FEBS Lett, 2000
- These results revealed some new targets of TGF beta  [ **TGFB1** ]  in endothelial cells, and differences in transcriptional regulation patterns between ALK 1  [ **ACVRL1** ]  and ALK  [ **ACVRL1** ]  5.  Ref: 12384983 J Cell Physiol, 2002
- We expressed the kinase domain of the TGF beta  [ **TGFB1** ]  type I receptor activin receptor like kinase ALK  [ **ACVRL1** ]  5 and the substrate, Smad3, and determined that SB 431542 is a selective inhibitor of Smad3 phosphorylation with an IC50 of 94 nM.  Ref: 12065755 Mol Pharmacol, 2002
- Here, we characterize a small molecule inhibitor SB 431542 that was identified as an inhibitor of activin receptor like kinase ALK  [ **ACVRL1** ]  5 the TGF beta  [ **TGFB1** ]  type I receptor.  Ref: 12065756 Mol Pharmacol, 2002
- In HUVEC, ALK 1  [ **ACVRL1** ]  was not detectable in the TGF beta1  [ **TGFB1** ]  or beta3 receptor complexes.  Ref: 10767348 Hum Mol Genet, 2000
- Newborn rat pups were treated with enalapril 30 mg kg per day or vehicle for 7 days, and kidneys were removed for Western blotting of TGF beta1  [ **TGFB1** ] , ALK 1  [ **ACVRL1** ] , ALK  [ **ACVRL1** ]  5, and TbetaRII, and for RT PCR of ALK  [ **ACVRL1** ]  5 and TbetaRII.  Ref: 12883979 Pediatr NephrolPediatr Nephrol, 2000
